# How anatomy shapes dynamics: a semi-analytical study of the brain at rest by a simple spin model

**DOI:** 10.3389/fncom.2012.00068

**Published:** 2012-09-20

**Authors:** Gustavo Deco, Mario Senden, Viktor Jirsa

**Affiliations:** ^1^Computational Neuroscience Group, Department of Information and Communication Technologies, Center for Brain and Cognition, Universitat Pompeu FabraBarcelona, Spain; ^2^Institució Catalana de la Recerca i Estudis Avançats, Universitat Pompeu FabraBarcelona, Spain; ^3^Faculty of Psychology and Neuroscience, Department of Cognitive Neuroscience, Maastricht UniversityMaastricht, Netherlands; ^4^Institut de Neurosciences des Systèmes UMR INSERM 1106, Aix-Marseille Université Faculté de MédecineMarseille, France

**Keywords:** computational neuroscience, fMRI modeling, ongoing activity, resting state, connectivity matrix

## Abstract

Resting state networks (RSNs) show a surprisingly coherent and robust spatiotemporal organization. Previous theoretical studies demonstrated that these patterns can be understood as emergent on the basis of the underlying neuroanatomical connectivity skeleton. Integrating the biologically realistic DTI/DSI-(Diffusion Tensor Imaging/Diffusion Spectrum Imaging)based neuroanatomical connectivity into a brain model of Ising spin dynamics, we found a system with multiple attractors, which can be studied analytically. The multistable attractor landscape thus defines a functionally meaningful dynamic repertoire of the brain network that is inherently present in the neuroanatomical connectivity. We demonstrate that the more entropy of attractors exists, the richer is the dynamical repertoire and consequently the brain network displays more capabilities of computation. We hypothesize therefore that human brain connectivity developed a scale free type of architecture in order to be able to store a large number of different and flexibly accessible brain functions.

## Introduction

Perceptions, memories, emotions, and everything that makes us human, demand the flexible integration of information represented and computed in a distributed manner. The human brain is structured into a large number of areas, in which information and computation are highly segregated, but then again are functionally integrated during normal brain function. Furthermore, human behavior entails a flexible task-dependent interplay between different subsets of brain areas in order to integrate them according to the corresponding goal-directed requirements. Nevertheless, the neuronal and cortical mechanisms governing the interactions and entrainment of different specialized brain areas for reaching that integration remain poorly understood. We contend that the functional and encoding roles of diverse neuronal populations across areas are subject to intra- and inter-cortical dynamics.

The main aim of this paper is to elucidate how the underlying anatomical structure shapes and determines global dynamics in a self-organizing manner. This will help us to understand the mechanisms underlying brain functions by complementing structural and activation-based analyses with dynamics. In particular, we expect to better comprehend the generation and interpretation of global and local spatio-temporal patterns of activity revealed at many levels of observations (fMRI, EEG, and MEG) in humans, and under task and resting (i.e., no stimulation and no task) conditions. An increasing number of experimental studies characterize the dynamics of spontaneous activity at rest with a variety of methods including EEG (Creutzfeldt et al., 1966), optical imaging (Kenet et al., 2003), single neuron recording (Engel, 2001), and fMRI (Biswal et al., 1995; Fox and Raichle, 2007). In particular, fMRI measures local changes in magnetic susceptibility (the blood oxygen level dependent, BOLD signal) caused by variations in the capillary concentration of deoxyhemoglobin, due to blood flow and blood volume increases in response to neuronal activation. Even at rest (i.e., in the absence of stimuli or a task), the spontaneous (intrinsic, not task-evoked) BOLD signal is characterized by slow oscillations (<0.1 Hz). It was noted over a decade ago that spontaneous BOLD signal fluctuations are temporally correlated (or coherent) between brain regions of similar functionality (Biswal et al., 1995; see Fox and Raichle, 2007 for a review). Regions showing high correlation at rest are said to be “functionally” connected; accordingly, this novel method of analysis of fMRI time series has been labeled either functional connectivity-by-MRI (fcMRI) or resting state-fMRI (rs-fMRI); finally, the ensuing networks of correlation are said to constitute resting state networks (RSNs). These RSNs may be functionally organized as dynamically competing systems both at rest and during different task conditions. In a series of papers, it has been shown that the cortical dynamics at rest can functionally be described as a dynamical system at a critical point; i.e., close to a critical point (Ghosh et al., 2008; Deco et al., 2009, 2011; Deco and Jirsa, 2012). At rest cortical areas exhibit pairwise synchronization following a power law scaling (Kitzbichler et al., 2009), which is also symptomatic of a dynamic system at the critical point. The RSNs are then multistable ghost attractors felt at that brink of bifurcation (Deco and Jirsa, 2012; Senden et al., 2012). A ghost attractor is a remnant of a fixed point that will emerge after bifurcation and manifests itself by slowing trajectories in phase space rather than capturing them (Gros, 2009; Friston and Ao, 2012). This implies that the system is at the brink of multistability but not yet multistable. Each attractor is explored but, in contrast to a multistable system, the system does not repeatedly settle in different attractors. Multistable ghost attractors allow for exploration of a functional repertoire without immediate execution of functions. In summary, the model of critical dynamics we have in mind can be described as follows: at rest, the brain operates in a critical regime, characterized by attractor ghosts or weak attractors in the Milnor sense. This leads to chaotic itinerancy—that may be augmented by random fluctuations and an exploration of multistable attractors (in the strong or classical sense). When a stimulus is encountered (or attentional set changes) one attractor becomes stable and is “selected” from a repertoire of latent attractors. Our focus in on how the dynamic repertoire (critical dynamics) is maintained at rest.

RSNs dynamics reflect the underlying anatomical connectivity between brain areas in a network (Bullmore and Sporns, 2009; Deco et al., 2011). Theoretical models allowed us to study the relation between anatomical structure and RSN dynamics (Honey et al., 2007; Ghosh et al., 2008; Deco et al., 2009). These models used realistic neuroanatomical information from the macaque cortex provided by the CoCoMac neuroinformatics database (Kötter, 2004), and from the human cortex provided by DTI/DSI (Diffusion Tensor Imaging/Diffusion Spectrum Imaging) techniques (Hagmann et al., 2008). In those models, the spatio-temporally structured functional connectivity evidenced in fMRI RSNs can hence be understood as the exploration of the brain's dynamic repertoire [see Ghosh et al. (2008)], which is captured here through the latent “ghost” multi-stable attractors at the edge of the bifurcation. Hagmann et al. (2008) demonstrated that the cortical architecture of the brain contains hubs. We speculate that precisely because of the existence of hubs, cortical networks are able to reflect a large number of RSNs. In other words, the large number of RSNs reflects the large number of “ghost” attractors structuring the noise under resting state conditions, i.e., the richness of the “dynamical repertoire” (Ghosh et al., 2008) that the noise around the trivial stable spontaneous state can explore.

Here, by using an analytically solvable Ising-Spin attractor model, we demonstrate that the emergence of structure in the fluctuations (and hence the dynamic repertoire) close to a critical point^1^ is richer if the network connectivity is scale free (as compared to other connection topologies including small world). Scale free networks can achieve maximal entropy in the multi-stable attractor region beyond that point. Additionally, we find that pairwise correlations between spins organized on a lattice representing human structural connectivity captures resting state functional connectivity; thus demonstrating the strong anatomical influence in the emergence of RSNs even in a system devoid of biologically realistic dynamics.

## Methods

### Empirical neuroanatomical and artificial connectivity matrices

**Figure 1:**
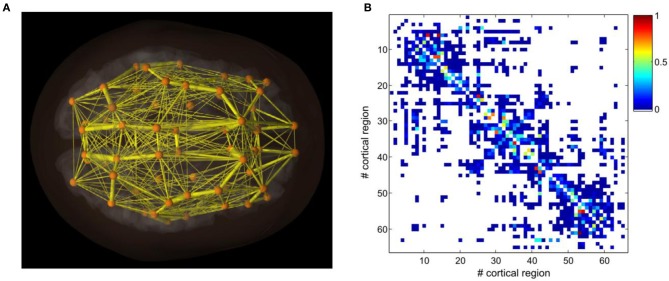
**Neuroanatomical connectivity data obtained by DSI and tractography after averaging across five human subjects (from Hagmann et al., 2008 and Honey et al., 2009). (A)** A three-dimensional reconstruction of connectivity patterns and spatial relations among cortical areas. **(B)** The structural connectivity matrix.

In this paper, we use a structural connectivity matrix composed of neuroanatomical connections between distinct brain areas in the human as well as a set of artificial connectivity matrices. Neuroanatomical connections in five human subjects were extracted by using DSI white matter tractography (Hagmann et al., 2008; Honey et al., 2009). This neuroanatomical matrix expresses the density, with which two different brain areas are connected through white matter fiber tracts. We used a segmented gray matter parcellation into 66 areas. The neuroanatomical matrix was finally averaged across the five human subjects. Figure 1A shows graphically the structure of the connectivity matrix by encoding the strengths of the different connections in a color map. The connectivity matrix is symmetric at the voxel level, due to the fact that tractography cannot distinguish the directionality of the fibers. Previous studies have shown that asymmetry may only play a role regarding the emergent network dynamics if the asymmetry is large (Knock et al., 2009; Jirsa et al., 2010). We order the different brain areas in the neuroanatomical connectivity matrix according to modules that have substantially denser connectivity within the module than with the complementary part of the network. Furthermore, homotopic regions in the two cerebral hemispheres were arranged symmetrically with respect to the center of the matrix. This reordering reveals graphically the small-world structure of brain networks through the presentation of clusters of varying size. In particular, the reordering of the connectivity matrix (see Figure 1A) shows the presence of clusters of nodes that are more connected inside than outside the cluster to which they belong, confirming previous observations (Bullmore and Sporns, 2009).

The small world architectures were generated with the classical method of Watts and Strogatz (1998) with a value *P* (probability of rewiring) of 0.25. The regular and random type of architectures were obtained with the same algorithm but for the extreme cases of *P* = 0 and *P* = 1, respectively. The scale free architectures were generated with the method of Albert and Barabási (2002).

### An analytically solvable ising-spin attractor model

We investigate the capabilities of different types of structural networks to sustain resting state activity by studying carefully the characteristics of their attractor landscapes by means of a reduced Ising-spin attractor model, which allows a thorough analytical investigation. The model is a network of stochastic binary units (“spins”).

**Figure 2:**
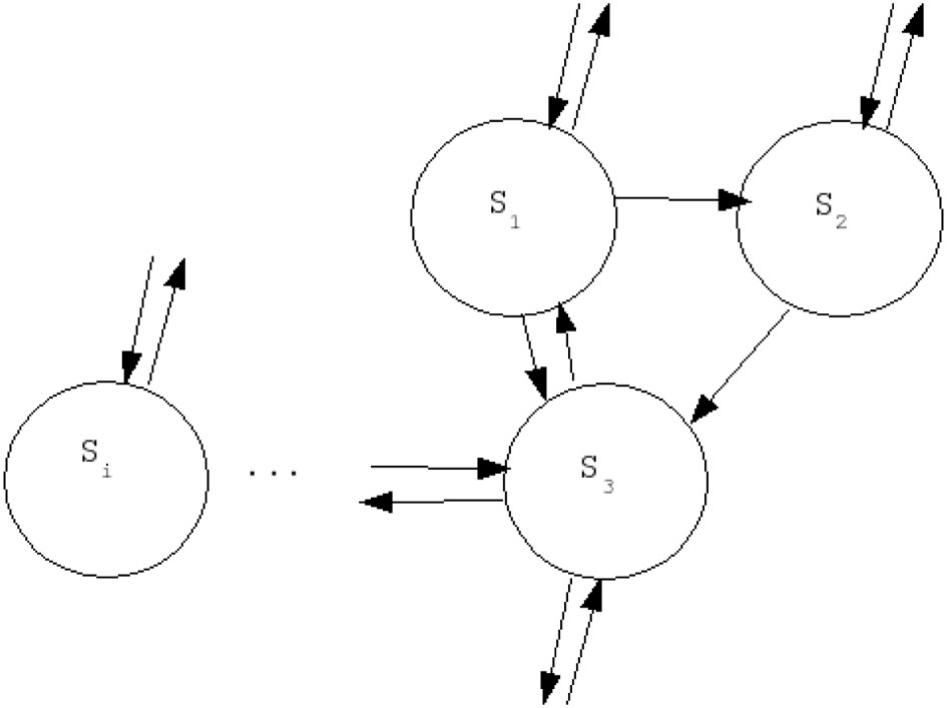
**Theoretical investigation of the activity in a simplified network of stochastic neural spins with Glauber dynamics: network architecture (see text for details)**.

Stochastic units describe the effect of thermal fluctuations in a system of Ising spins in the presence of a field. In statistical physics this property describes a so-called Glauber dynamics (Glauber, 1963). Each unit will be associated with one specific node (“cortical brain area”) and they will be symmetrically coupled according to the connectivity matrix associated with the neuroanatomical network to be studied. We consider here different types of artificial structural networks, namely: regular, random, small world and scale free; as well as a neuroanatomical connectivity matrix obtained by DSI. Let us denote by *S*_*i*_ the state of the spin unit of the node *i*, and by *C*_*ij*_ the connectivity matrix associated with the particular structure of the network. The existence of a connection is encoded by 1 and its absence by 0, as usual (see Figure 2).

The Glauber dynamics of this network can be described by the following equations:

(1) $$\{\begin{array}{l} S_{i}=1\text{ with probability }p_{i}\;\\ S_{i}=0\text{ with probability 1-}p_{i} \end{array}$$

where *p*_*i*_

(2) $$p_{i}=g\left(W\sum_{j}C_{ij}S_{j}-\theta \right)$$

and

(3) $$g(X)=\frac{\text{1}}{\text{1+}e^{-\varepsilon X}}$$

In equation 2, θ is the threshold, and *W* is a parameter regulating the global coupling strengths between the nodes. In our simulations, we used: θ = 12 and *W* = 1. The parameter ε in equation 3 denotes an inverse temperature. Let us further label the global state of all units for a given configuration by a superindex α. For symmetric connections, the Boltzmann-Gibbs distribution giving the probability of finding the network in a specific state *S*^α^ can be expressed analytically by

(4) $$P^{\alpha }=\frac{e^{-\varepsilon H^{\alpha }}}{Z},$$

where *Z* is the partition function defined by

(5) $$Z=\sum_{\alpha }e^{-\varepsilon H^{\alpha }}$$

and *H*^α^ is the energy function

(6) $$H^{\alpha }=\frac{1}{2}\theta \sum_{i}S_{i}^{\alpha }-\frac{1}{2}W\sum_{i,j}S_{i}^{\alpha }S_{j}^{\alpha }$$

The probability *P*^α^ gives the probability of finding the configuration *S*^α^. Therefore, in order to describe the attractor landscape of the spin network, we can characterize the existence and probability of each possible attractor (here corresponding to a specific configuration *S*^α^) by the entropy of the system, which can be derived analytically, yielding:

(7) $$E=\sum_{\alpha }P^{\alpha }logP^{\alpha }=\frac{\sum_{\alpha }\varepsilon H^{\alpha }e^{-\varepsilon H^{\alpha }}}{Z}\text{+ log}Z$$

For the artificial connectivity matrices we restricted the size of the network to a maximum of 20 due to computational restrictions in the calculation of the entropy (the sum over all configurations increase exponentially with the size of the network and for each network 100 instances are considered). To achieve computational feasibility with regard to empirical structural connectivity we split the connectivity matrix into a left and right hemisphere of 33 nodes each. Furthermore, we parallelize the calculation of entropy for empirical SC by distributing chunks of the 2^33^ states across GPU processors.

We, furthermore, investigate the degree of association between individual spins. For the artificial matrices we used mutual information. The mutual information is an excellent measure for pairwise association, but is usually very difficult to calculate, because it requires the estimation of the underlying marginals and joint probabilities. Nevertheless, in the Ising-spin system, because the probability of each state is analytically given (Equation 3), we can calculate the mutual information exactly. We sampled mutual information between all pairs of nodes and over 100 random instantiations of scale free networks containing 20 nodes and 38 edges. For human structural connectivity we calculate model functional connectivity by estimating pairwise correlations of spin activity^2^ across all global states.

## Results

In a previous study (Deco and Jirsa, 2012), we have shown that the emergence of RSNs is due to structured noise fluctuations around the trivial equilibrium state induced by the presence of latent “ghost” multi-stable attractors (the dynamic repertoire) at the edge of the bifurcation. Here, we study the characteristics of that relevant bifurcation for different types of connectivity analytically by investigating how the entropy of the Ising-spin network evolves as a function of global coupling strength.

**Figure 3:**
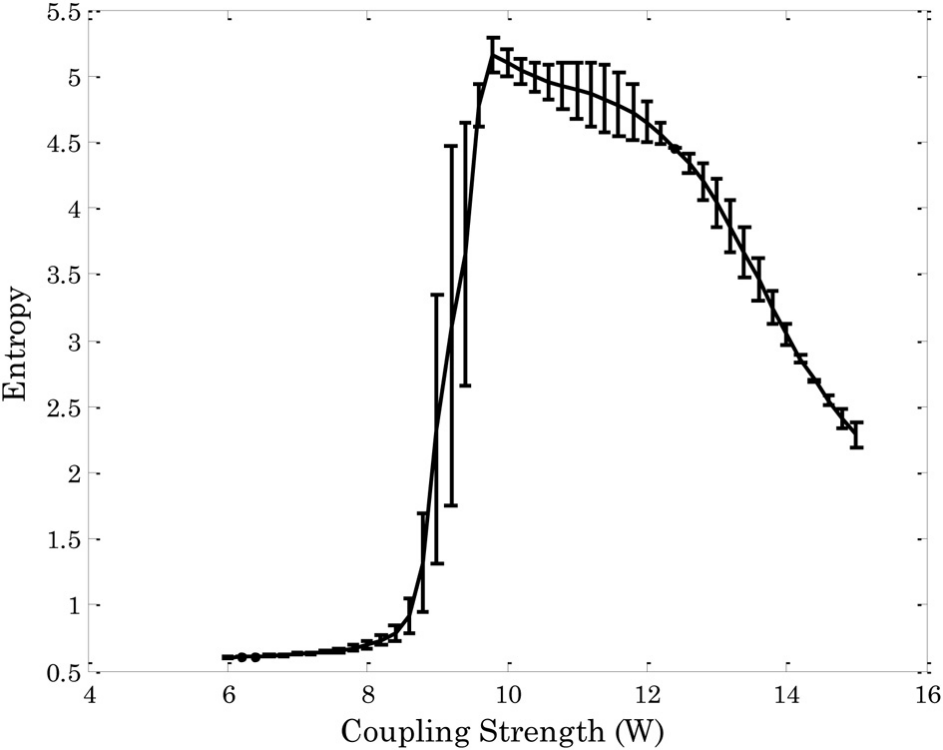
**Entropy of the attractors in an Ising-spin network reflecting human structural connectivity as a function of the global coupling strength.** The rapid increase of entropy corresponds to the bifurcation. At the brink of this region resting state networks emerge.

**Figure 4:**
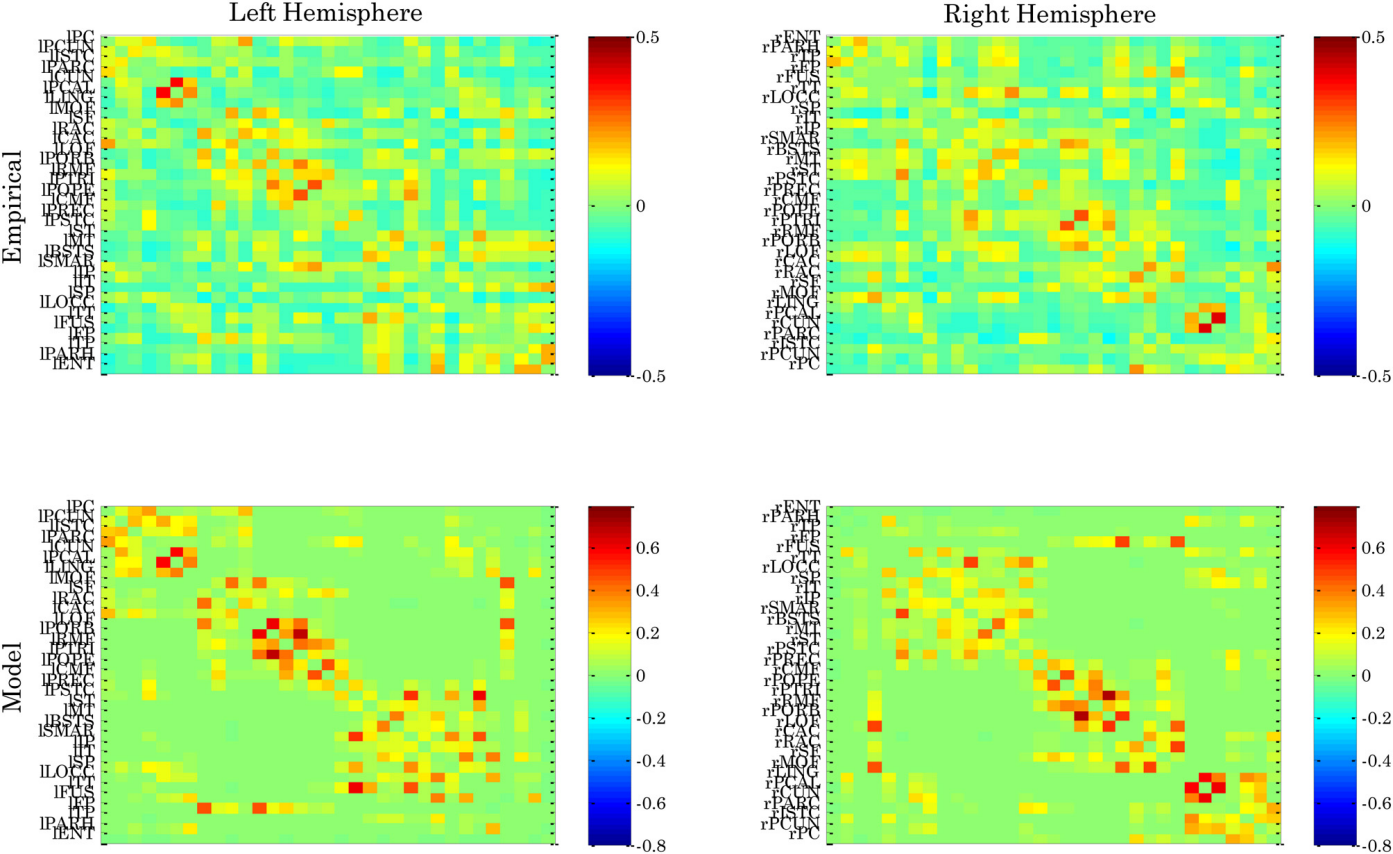
**Functional connectivity observed for the left and right hemisphere of empirical resting state data and spin glass model.** The ordering of cortical areas corresponds to that of the underlying neuroanatomical connectivity matrix.

Figure 3 shows the entropy of the attractors^3^ in an Ising-spin network reflecting human structural connectivity. For small values of the coupling *W* the architecture shows low entropy corresponding to the existence of one unique trivial state where all spins are 0. In the same form, for large values of the coupling *W* the architecture shows again low entropy corresponding now to the existence of one unique “epileptoform” state where all the spins are 1. For intermediate values of the coupling strength many attractors can coexist with different probabilities, so that the entropy increases to a maximum value. The relevant region for the resting state is at the edge of the bifurcation separating the trivial spontaneous state (all spins 0) and the emergence of attractors corresponding to higher activity (i.e., 1) in some other nodes. In other words, the region of *W* where the entropy starts to increase evidences the relevant bifurcation. Indeed, at this bifurcation point the functional connectivity as obtained from the pairwise correlation in the Ising-spin system closely resembles empirical functional connectivity (Figure 4). Specifically, the correlation between the pattern of pairwise correlations observed in model and empirical FC is 0.58 for the right and 0.56 for the left hemisphere, respectively.

**Figure 5:**
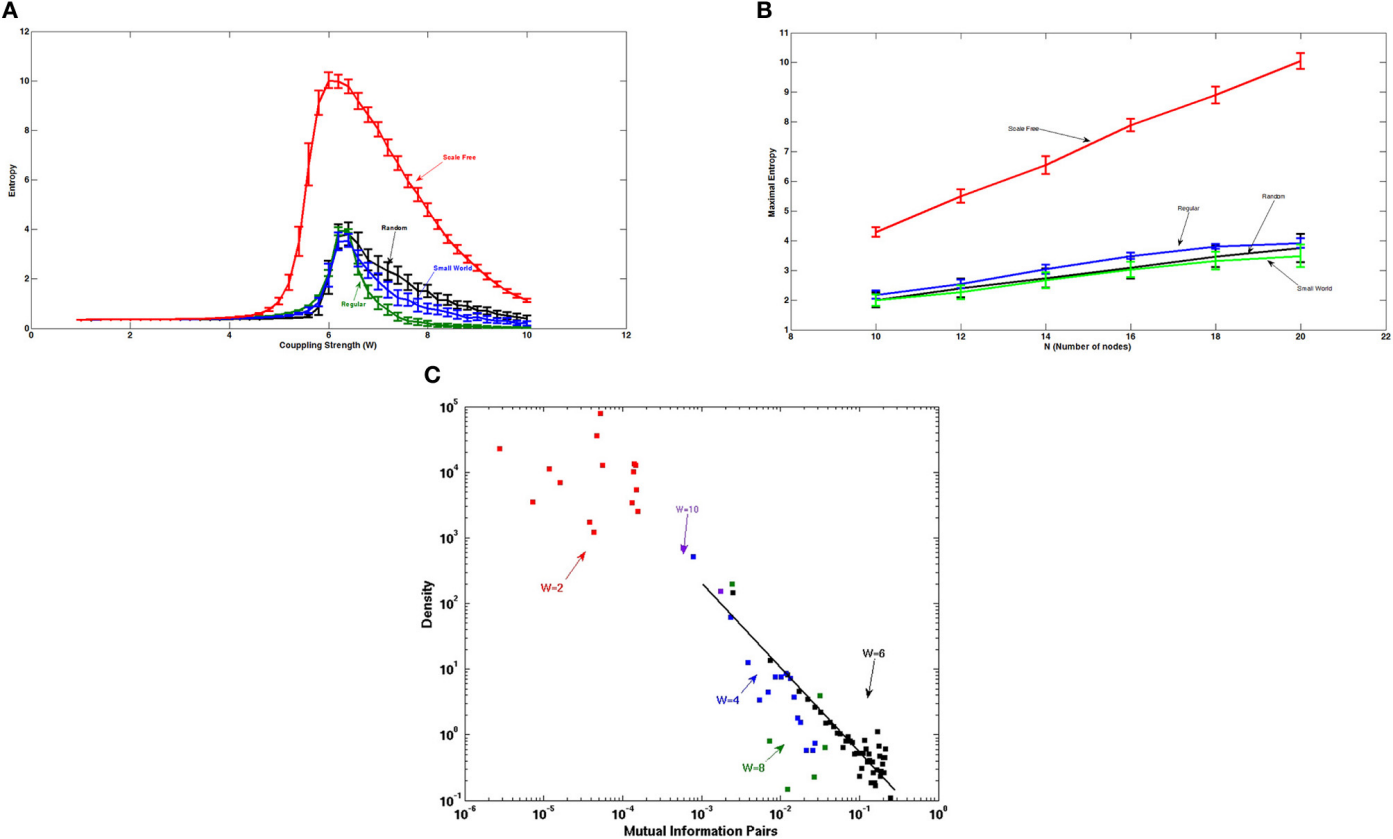
**(A)** Entropy of the attractors of Ising-spins networks of 20 nodes and 38 edges as a function of the global coupling strength. Scale free architectures are able to sustain a much richer dynamical repertoire as evidenced by the larger maximal value of the entropy of the attractors than the one corresponding to the small world, regular and random networks. **(B)** Evolution of the maximal entropy value obtained with the different architectures as a function of the number of nodes. The scale free network shows a much faster increase of the maximal entropy than small world type of networks. Importantly, the number of connections each node makes remains unchanged implying that sparsity of the network does not influence the results. **(C)** Distribution of the pair correlations for scale free networks of 20 nodes and 38 edges, and for different coupling values. At the edge of the bifurcation, i.e., when the entropy is maximal, the distribution of the pair correlations shows a power law.

Figure 5A shows the entropy of the attractors of Ising-spin networks of artificial networks consisting of 20 nodes and 38 connections as a function of the global coupling strength *W*. Here, we contrast the entropy for different underlying topographical structural connections. The maximum of the entropy obtained for the different network architectures is different. Small world architectures, including different level of small-worldness, from the regular to the random, show a similar maximal value of the entropy. In contrast, the scale free architecture shows a much larger maximal value of the entropy suggesting that this type of structure can sustain a much richer number of relevant attractors. This is due to the fact that scale free topologies contain hubs, i.e., nodes with a larger number of connections, and therefore allow the formation of widely distributed attractors. The higher the entropy, the higher is the number of “ghost” attractors that efficiently structure the fluctuations of the system at the edge of the bifurcation for building the resting state and therefore the higher will be the resulting number of RSNs.

Figure 5B shows the evolution of the maximal entropy obtained with the different architectures as a function of the number of nodes and for a matched number of edges. The number of attractors that a scale free network can store increases much faster than the amount of attractors that small world type of networks can store. This means that scale free networks are extremely efficient for computation.

The power-law distribution of pair correlations at the edge of the bifurcation that we have shown in a previous study (Deco and Jirsa, 2012) is also evidenced in the Ising spin system. Figure 4 shows the distribution of the pair associations as calculated with the mutual information between two different nodes. Figure 5C shows that only at the edge of the bifurcation, i.e., when the entropy is maximal, the distribution of the pair associations shows a power law. For the extreme cases of small or large coupling *W*, the whole system is highly correlated because there is practically only one state (all spin equal 0 or 1, respectively) and therefore there is no variability and the mutual information is very small, consequently the whole distribution is shifted to the left part of the graph. Intermediate cases approach broader distributions, but a power law tail is only obtained for the coupling corresponding to the maximum entropy value.

## Discussion

In this paper, we have demonstrated using analytically solvable Ising-model that the number of attractors in a network model is linked to its dynamical repertoire. This link is quantified by the entropy measure. The interpretation of the set of attractors as a dynamic repertoire (see Ghosh et al., 2008) offers exciting insights into brain function and sheds light on the functional role of the resting state. At rest the brain network seems to operate close to a critical point as illustrated here and in previous studies (Deco and Jirsa, 2012; Senden et al., 2012). It is, however, not possible to make a straightforward judgment as to whether this particular system is supercritical or subcritical. Subcriticality would imply the existence of multistable attractors which are perturbed and hence explored due to noise. Supercriticality would imply a resting “ground” state within which the presence of latent ghost attractors can be felt. This is what we have observed in previous studies (Deco and Jirsa, 2012; Senden et al., 2012). Therefore, although the exact nature of the system remains an open and intriguing question, we currently conceptualize the brain at rest as a supercritical system. That is, close to the critical point, a set of multistable “ghost” attractors is available. Assuming this set of attractors to be functionally meaningful, the computational brain mechanism to invoke a particular function involves destabilization of the resting state and stabilization of the desired attractor state. Since the states already exist in the dynamic repertoire of the brain network, they do not need to be created, which suggests the advantage of rapid computation of a specific brain function through stabilization of one of its attractors. Consequently, the more entropy of attractors exists, the richer is the dynamical repertoire and therefore the brain network displays more capabilities of computation.

Previous studies of network models (Deco and Jirsa, 2012; Senden et al., 2012) have found a maximum of five attractor states in the network's repertoire. Empirical studies (such as Damoiseaux et al., 2006) counted eight robust patterns to be present. At this point, the precise number itself is not relevant, but the fact that a small number of patterns is favored is interesting in itself. What is it that distinguishes these network patterns from the multitude of all possible patterns? First of all, as we have shown here and in previous work, the whole of the network states defines a dynamic repertoire of the brain network that is inherently present in the neuroanatomical connectivity. Further, empirical studies have demonstrated that there seems to be a large overlap of the RSNs with network patterns known from task specific activations (Damoiseaux et al., 2006; Smith et al., 2009). In combination, these two findings underscore the potential relevance of the attractor states stored in the brain network for the selection of a functional system. They leaven open, however, how the actual functional specificity within such a system is accomplished. This question can and is more appropriately addressed by systems neuroscience.

Our analyses argue that a richer dynamical repertoire arise when the system is integrated on a scale-free network in comparison to a random, regular or small world network (all of which have near uniform degree distribution). This argues that an increased dynamical repertoire (and hence greater adaptive range of responses) may have exerted selective pressure during evolution toward a scale-free node arrangement. This is also evident from the betweenness centrality observed in both human as well as scale free connectivity matrices. Specifically, the distribution of betweenness centrality significantly follows a power law in human structural connectivity data we used in the present study (γ = 2.81 *p* = 0.7, estimated using the procedure described by Clauset et al., 2007,^4^). The same is true for scale free matrices (Goh et al., 2001). Because it measures the traffic going through a node, betweenness centrality is a complementary measure of the importance of a node to the degree of connectivity of that node. However, in empirical, spatially embedded networks, scale-free degree distribution is limited by the impracticality of creating nodes with connections to almost all other brain regions through an upper bound of achievable synaptic density. Recent empirical analyses of human tractographic data suggest that the degree distribution may be heavy tailed, but is strongly truncated and spans less than two orders of magnitude (Zalesky et al., 2010). Hence the two competing forces (greater range of dynamic responses versus spatial constraints on local wiring density) may find their balance in the emergence of an exponentially truncated power law degree distribution.

In conclusion, we showed here that the brain network builds its neuroanatomical connections in an approximately scale free type of architecture, which is able to store a large number of different and flexibly accessible brain functions. The numerous brain functions are evidenced indirectly under resting state conditions by the generation of a large diversity of networks reflecting different ways of structured fluctuations, i.e., by the RSNs. All these mechanisms will be studied in future works.

### Conflict of interest statement

The authors declare that the research was conducted in the absence of any commercial or financial relationships that could be construed as a potential conflict of interest.

## Footnotes

^1^We do not refer to phase transitions since on a finite lattice an Ising system has finite degrees of freedom and the concept of phase transitions applies to systems with infinite degrees of freedom.

^2^For each global state the activity of spins initially equal to 0 is estimated as the hyperbolic tangent of the weighted input from active neighbors.

^3^In our treatment, we have interpreted the entropy of the ensemble distribution over states our system can occupy as entropy over attractors. Strictly speaking, this entropy is simply over the states that are constituted by attracting sets, which are random. The generalization of the concept of an attractor in the classical (deterministic) sense in the setting of random dynamical systems—of the sort we are modeling—calls upon the notion of a pullback attractor and represents a fairly well developed measure-theoretic formulation of dynamical systems (see Crauel et al., 1997). We have assumed here, heuristically, that the notions of bifurcations and multistability have their counterparts in the random dynamical system formulation.

^4^The authors use a combination of maximum-likelihood fitting methods and goodness-of-fit tests based on likelihood ratios and the Kolmogorov–Smirnov statistic to quantify power-law behavior in empirical data.

## References

[B1] Albert R. Barabási A. L. (2002). Statistical mechanics of complex networks. Rev. Mod. Phys. 74, 47–97

[B2] Biswal B. Yetkin F. Z. Haughton V. M. Hyde J. S. (1995). Functional connectivity in the motor cortex of resting human brain using echo-planar MRI. Magn. Reson. Med. 34, 537–541 8524021 10.1002/mrm.1910340409

[B3] Bullmore E. Sporns O. (2009). Complex brain networks: graph theoretical analysis of structural and functional systems. Nat. Rev. Neurosci. 10, 186–198 10.1038/nrn2575 19190637

[B5a] Clauset A. Shalizi C. Newman M. (2007). Power-law distributions in empirical data. SIAM Rev. 51, 661–703

[B4] Crauel H. Debussche A. Flandoli F. (1997). Random attractors. J. Dyn. Differ. Equ. 9, 307–341

[B5] Creutzfeldt O. D. Watanabe S. Lux H. D. (1966). Relations between EEG phenomena and potentials of single cortical cells. I. Evoked responses after thalamic and epicortical stimulation. Electroencephalogr. Clin. Neurophysiol. 20, 1–18 4161317 10.1016/0013-4694(66)90136-2

[B6] Damoiseaux J. S. Rombouts S. A. Barkhof F. Scheltens P. Stam C. J. Smith S. M. Beckmann C. F. (2006). Consistent resting-state networks across healthy subjects. Proc. Natl. Acad. Sci. U.S.A. 103, 13848–13853 10.1073/pnas.0601417103 16945915 PMC1564249

[B7a] Deco G. Jirsa V. K. (2012). Ongoing cortical activity at rest: criticality, multistability, and ghost attractors. J. Neurosci. 32, 3366–3375 10.1523/JNEUROSCI.2523-11.2012 22399758 PMC6621046

[B7] Deco G. Jirsa V. McIntosh A. R. (2011). Emerging concepts for the dynamical organization of resting-state activity in the brain. Nat. Rev. Neurosci. 12, 43–56 10.1038/nrn2961 21170073

[B8] Deco G. Jirsa V. McIntosh A. R. Sporns O. Kotter R. (2009). Key role of coupling, delay, and noise in resting brain fluctuations. Proc. Natl. Acad. Sci. U.S.A. 106, 10302–10307 10.1073/pnas.0901831106 19497858 PMC2690605

[B9] Engel A. K. (2001). Dynamic predictions: oscillations and synchrony in top-down processing. Nat. Rev. Neurosci. 2, 704 10.1038/35094565 11584308

[B10] Friston K. Ao P. (2012). Free energy, value, and attractors. Comput. Math. Methods Med. 2012, 1–27 10.1155/2012/937860 PMC3249597 22229042

[B11] Fox M. D. Raichle M. E. (2007). Spontaneous fluctuations in brain activity observed with functional magnetic resonance imaging. Nat. Rev. Neurosci. 8, 700–711 10.1038/nrn2201 17704812

[B12] Ghosh A. Rho Y. McIntosh A. R. Kotter R. Jirsa V. K. (2008). Noise during rest enables the exploration of the brain's dynamic repertoire. PLoS Comput. Biol. 4:e1000196 10.1371/journal.pcbi.1000196 18846206 PMC2551736

[B13] Glauber R. J. (1963). Time-dependent statistics of the ising model. J. Math. Phys. 4, 294

[B14] Goh K.-I. Kahng B. Kim D. (2001). Universal behavior of load distribution in scale-free networks. Phys. Rev. Lett. 87, 278701–278704 10.1103/PhysRevLett.87.278701 11800921

[B15] Gros C. (2009). Cognitive computation with autonomously active neural networks: an emerging field. Cogn. Comput. 1, 77–90

[B16] Hagmann P. Cammoun L. Gigandet X. Meuli R. Honey C. J. Wedeen V. J. Sporns O. (2008). Mapping the structural core of human cerebral cortex. PLoS Biol. 6:e159 10.1371/journal.pbio.0060159 18597554 PMC2443193

[B17] Honey C. J. Kotter R. Breakspear M. Sporns O. (2007). Network structure of cerebral cortex shapes functional connectivity on multiple time scales. Proc. Natl. Acad. Sci. U.S.A. 104, 10240–10245 10.1073/pnas.0701519104 17548818 PMC1891224

[B18] Honey C. J. Sporns O. Cammoun L. Gigandet X. Thiran J. P. Meuli R. Hagmann P. (2009). Predicting human resting-state functional connectivity from structural connectivity. Proc. Natl. Acad. Sci. U.S.A. 106, 2035–2040 10.1073/pnas.0811168106 19188601 PMC2634800

[B19] Jirsa V. Sporns O. Breakspear M. Deco G. McIntosh A. R. (2010). Towards the Virtual brain: network modeling of the intact and the damaged brain. Arch. Ital. Biol. 148, 189–205 21175008

[B20] Kenet T. Bibitchkov D. Tsodyks M. Grinvald A. Arieli A. (2003). Spontaneously emerging cortical representations of visual attributes. Nature 425, 954–956 10.1038/nature02078 14586468

[B21] Kitzbichler M. Smith M. Christensen S. Bullmore E. (2009). Broadband criticality of human brain networks synchronization. PLoS Comput. Biol. 5:e1000314 10.1371/journal.pcbi.1000314 19300473 PMC2647739

[B22] Knock S. A. McIntosh A. R. Sporns O. Kötter R. Hagmann P. Jirsa V. K. (2009). The effects of physiologically plausible connectivity structure on local and global dynamics in large scale brain models. J. Neurosci. Methods 183, 86–94 10.1016/j.jneumeth.2009.07.007 19607860

[B23] Kötter R. (2004). Online retrieval, processing, and visualization of primate connectivity data from the CoCoMac database. Neuroinformatics 2, 127–144 10.1385/NI:2:2:127 15319511

[B25] Senden M. Goebel R. Deco G. (2012). Structural connectivity allows for multi-threading during rest: the structure of the cortex leads to efficient alternation between resting state exploratory behavior and default mode processing. Neuroimage 60, 2274–2284 10.1016/j.neuroimage.2012.02.061 22394674

[B26] Smith S. M. Fox P. T. Miller K. L. Glahn D. C. Fox P. M. Mackay C. E. Filippini N. Watkins K. E. Toro R. Laird A. R. Beckmann C. F. (2009). Correspondence of the brain's functional architecture during activation and rest. Proc. Natl. Acad. Sci. U.S.A. 106, 13040–13045 10.1073/pnas.0905267106 19620724 PMC2722273

[B27] Watts D. J. Strogatz S. H. (1998). Collective dynamics of ‘small-world’ networks. Nature 393, 409–410 9623998 10.1038/30918

[B28] Zalesky A. Fornito A. Harding I. H. Cocchi L. Yücel M. Pantelis C. Bullmore E. T. (2010). Whole-brain anatomical networks: does the choice of nodes matter? Neuroimage 50, 970–983 10.1016/j.neuroimage.2009.12.027 20035887

